# The association between obesity and blood pressure in Thai public school children

**DOI:** 10.1186/1471-2458-14-729

**Published:** 2014-07-18

**Authors:** Penmat Sukhonthachit, Wichai Aekplakorn, Chatrapa Hudthagosol, Chutima Sirikulchayanonta

**Affiliations:** 1 Department of Nutrition, Faculty of Public Health, Mahidol University, 420/1 Rajvithi Road, Rajthevi distric, Bangkok 10400, Thailand; 2 Department of Community Medicine, Faculty of Medicine, Ramathibodi Hospital, Mahidol University, 270 Rama VI Road, Rajthevi district, Bangkok 10400, Thailand

**Keywords:** Child obesity, High blood pressure, School children, Waist circumference

## Abstract

**Background:**

The prevalence of obesity has substantially increased in the past 3 decades in both developed and developing countries and may lead to an increase in high blood pressure (BP) at an early age. This study aimed to determine the prevalence of obesity and its association with blood pressure among primary school children in central Thailand.

**Methods:**

A cross-sectional study was conducted in two public schools in Bangkok in 2012. A total of 693 students (317 boys and 376 girls) aged 8–12 years participated voluntarily. Anthropometric measurements of weight, height, waist circumference (WC) and BP were collected. Fasting venous blood samples were obtained for biochemical analysis of fasting plasma glucose (FPG) and lipid parameters. Child nutritional status was defined by body mass index (BMI) for age based on the 2000 Center for Diseases Control and Prevention growth charts. The cutoff for abdominal obesity was WC at the 75 percentile or greater. Hypertension was defined according to the 2004 Pediatrics US blood pressure reference. Multinomial logistic regression was used to examine the relationship between high BP and obesity after controlling for other covariates.

**Results:**

The prevalence of obese children was 30.6% for boys and 12.8% for girls (mean prevalence 20.9%). Pre-hypertension (Pre-HT) was 5.7% and 2.7% for boys and girls and hypertension (HT) was 4.7% for boys and 3.2% for girls, respectively. Children with pre-HT and HT had significantly higher body weight, height, WC, BMI, SBP, DBP, TG, and TC/HDL-C levels but lower HDL-C levels than those children with normotension. After controlling for age, sex, glucose and lipid parameters, child obesity was significantly associated with pre-HT and HT (odds rations (ORs) = 9.00, 95% CI: 3.20-25.31 for pre-HT and ORs = 10.60, 95% CI: 3.75-30.00 for HT). So also was WC (abdominal obesity) when considered alone (ORs = 6.20, 95% CI: 2.60-14.81 for pre-HT and ORs = 13.73, 95% CI: 4.85-38.83 for HT) (p-value < 0.001).

**Conclusions:**

Obesity among school children was positively associated with higher BP. Prevention of childhood obesity should be strengthened to prevent the risk of early high BP including cardiovascular risk factors.

## Background

Childhood obesity based on body mass index (BMI) has dramatically increased over the past 3 decades in both developed and developing countries [1] together with many of its health consequences such as dyslipidemia, high blood pressure (BP), abnormal plasma glucose levels and metabolic disorders [2-4]. These are the cardiovascular risk factors that start from childhood and may continue into adulthood [5] to become a public health concern in various countries [6]. In Thailand, studies of obesity prevalence in children aged 6–12 years from a National Health Examination Survey using weight-for-height criteria [7] revealed that overweight and obese children increased nationally from 5.8% in 1997 to 6.7% in 2001 and then became substantially higher at 8.7% in 2010, with the highest regional prevalence (14.8%) in Bangkok [8]. In 2004, the Bright and Healthy Thai Kid (BAHT) project was launched in 4 Bangkok public schools with the aim of reducing childhood obesity, and the reported baseline prevalence of obesity and high blood cholesterol in the study group was 19.3% and 40%, respectively [2]. Several studies have proposed an association between obesity and high blood pressure, including increased cardiovascular risk factors [9,10]. However, current data in Thailand regarding this association are limited, particularly for children. This study aimed to determine the prevalence of obesity and its association with high blood pressure among primary school children.

## Methods

### Research design

This was a cross-sectional study that was conducted during May-July 2012 in Bangkok primary public schools in central Thailand.

### School selection

From four public schools in Bangkok Metropolis under the Office of Basic Education (OBEC) and included in the earlier BAHT project, two were randomly selected. Both schools were coeducational, and had similar demographics for number of students (more than 700 students), gender, school environment, family socioeconomic status (low to middle class) [2] and parental support. Moreover, administrators of both schools were willing to support and participate in the study.

### Participants

Participants were students in grades 3–6 aged 8–12 years during the 2012 academic year and they voluntarily joined this study. The sample size (n) was determined by the Cochran formula [11] :

$$n=\frac{Z^{2}\mathrm{pq}}{e^{2}}$$

where *Z* is the standard Z score at α/2 = 0.025, Z = 1.96, where the proportion (p) of childhood obesity was estimated from a previous study [2] in primary schools as 0.193 so that q was calculated as q = 1 – p = 0.807 and where the error e at 0.05 was taken from the same study. This yielded a total minimum sample of 240 children.

Informed consent and assent forms were obtained from 63% (a total of 693 students with 317 boys and 376 girls) and their parents. The proposal was reviewed and approved by the Institutional Review Board, Faculty of Public Health, Mahidol University (Proof No. 100/2555 MUPH 2012–132).

### Data collection

#### Anthropometric assessment of weight, height and waist circumference

Weight was measured in kilograms with one decimal point using an electronically calibrated scale (Seca, German) and height was measured in centimeters with one decimal point using a calibrated stadiometer (Microtoise) according to standard measurement procedures [12]. Age and sex specific BMI percentiles for each child were calculated based on the 2000 Centers for Disease Control and Prevention growth charts [13]. Child nutritional status was categorized based on BMI percentile criteria as follows: < 5^th^ percentile for underweight, ≥ 5^th^ but < 85^th^ percentiles for normal, ≥ 85^th^ but < 95^th^ percentiles for overweight: and ≥ 95^th^ percentile for obese, respectively [13] (see details in Additional file 1: 1.1 Child nutritional status). WC was measured at the umbilicus level at ex-hale using a non-elastic tape with participants in a standing position [14]. The mean of three measurements was used for this analysis. The WC cutoff for age- and sex specific abdominal obesity was defined as the 75^th^ percentile (P_75_) or greater (Additional file 2).

### Clinical assessment of blood pressure (BP)

BP was measured by two trained research assistants in the morning on the left arm of participants in a sitting position after resting at least 5 minutes [15]. The cuff size was based on arm length and circumference of the upper arm of participants (17 × 22 and 22 × 32 cm). Three measurements at intervals 2–5 minutes apart were obtained by using an automatic blood pressure monitor “Microlife BP-A100 model” (Microlife AG, Widnau, Switzerland) [16]. An average of the second and third values was used in the analysis [17].

SBP and DBP percentiles were calculated according to sex, age and height percentile based on the 2000 CDC growth chart and on the fourth report on diagnosis, evaluation, and treatment of high BP in children and adolescents from the national high blood pressure education program working group on high BP in children and adolescents [18]. BP status was classified according to SBP and/or DBP as follows: < 90^th^ percentile for normal SBP and/or DBP, ≥ 90^th^ but < 95^th^ percentile for pre-hypertension, and ≥ 95^th^ percentile for hypertension [18] (see details in Additional file 1: 1.2 Child blood pressure classification).

### Biochemical assessment

Participants were instructed to fast after 20:00 h. (10–12 hours). A fasting venous blood sample of 5–7 milliliters from each participant was collected in the morning before breakfast. Blood samples were kept in an ice-box (temperature 4°C) and sent to the Office of Public Health and Environment Technology Services (OPHETS) laboratory, Faculty of Public Health, Mahidol University for automated analysis of FPG and lipid profiles (TC, TG, LDL-C, HDL-C) by the enzymatic method (Beckman Coulter, AU680). The average coefficient of variation for within runs/between days in precision of FPG, TC, TG and HDL-C were 1.05%/2.3%, 1%/1.45%, 1.2%/2.15% and 0.65%/1.4%, respectively.

### Statistical analyses

All the data were analyzed using Predictive Analytics Software for Windows (PASW) version 18. Continuous variables (age, weight, height, BMI, WC, SBP, DBP, FPG, TC, TG, LDL-C, HDL-C and TC/HDL-C) were used to describe general characteristics of children with mean and standard deviation (SD) and the means were compared for gender differences using a Student t-test. The categorical variables for age- and sex-specific BMI and WC as numbers and percentages were compared using χ^2^ analysis. Analysis of covariance (ANCOVA) was used to compare the mean difference of anthropometric measurements, blood sugar level and lipid parameters among BP status. Multiple regression was used to examine the association of SBP and DBP with BMI and WC, controlling for other covariates including age, sex, plasma glucose and lipid parameters. Multinomial logistic regression was used to examine the association between BP status (0 = normotension, 1 = pre-hypertension, and 2 = hypertension) as dependent variables with nutritional status adjusted for the covariate variables including age, sex, plasma glucose and lipid levels. Odds ratios and 95% confidence intervals (95% CI) were determined. Differences with p-values ≤ 0.05 were considered to be statistically significant.

## Results

**Figure 1:**
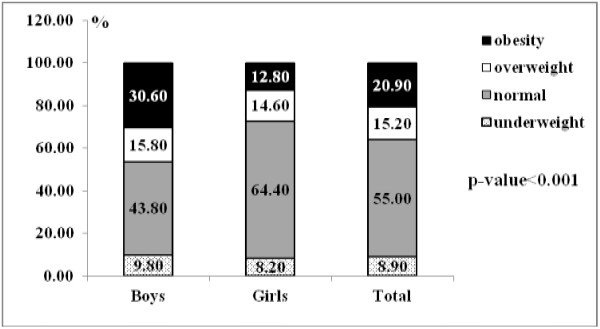
Nutritional status using BMI for age among school children.

**Figure 2:**
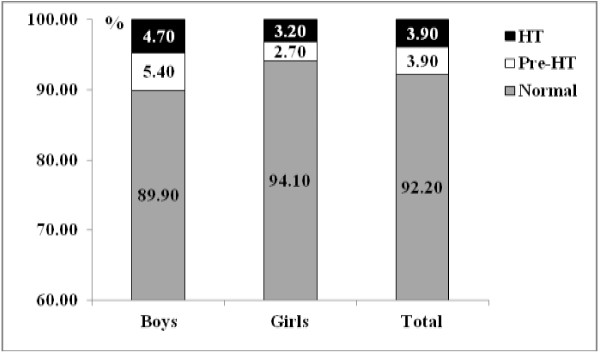
Prevalence of pre-hypertension and hypertension among school children.

The study included a total of 693 participants (317 boys and 376 girls) whose mean ages were 10.29 (boys) and 10.39 (girls). General characteristics, anthropometric data, FPG and lipid profile of the participants are presented in Table 1. Boys had significantly higher BMI, WC, SBP and FPG levels than girls (p-values < 0.001). However, girls had significantly higher TC, TG, LDL-C, and TC/HDL-C levels than boys (p-values < 0.001). There were no statistically significant differences in average height, DBP and HDL-C levels. Prevalence of obesity was 30.6% in the boys and 12.8% in the girls (mean for both 20.9%) (Figure 1). Prevalence of pre-HT was 5.4% for boys and 2.7% for girls and HT was 4.7% and 3.2% for boys and girls, respectively (Figure 2). In Table 2, the prevalence of pre-HT and HT was 13.1% and 13.8% for obese children and 10.5% and 12.9%, for children when considering WC (abdominal obesity) alone. All these prevalences were significantly higher than those of non-obese children (p-value < 0.001).

**Table 1:** Characteristics (mean ± SD) of school children on anthropometric measures, blood pressure, plasma glucose and lipid profile

**Variables**	**Total (N = 693)**	**Male (n = 317)**	**Female (n = 376)**	**p-value**
	$\bar{X}$ **± SD**	$\bar{X}$ **± SD**	$\bar{X}$ **± SD**	
**Age (years)**	10.34 ± 1.15	10.29 ± 1.16	10.39 ± 1.14	0.24
**Weight (kg)**	38.82 ± 12.50	39.85 ± 13.71	37.95 ± 11.33	0.05
**Height (cm)**	140.88 ± 10.33	140.09 ± 9.96	141.55 ± 10.60	0.064
**BMI (kg/m**^**2**^**)**	19.19 ± 4.47	19.85 ± 4.90	18.63 ± 4.00	< 0.001
**WC (cm)**	70.35 ± 12.05	72.28 ± 13.43	68.73 ± 10.50	< 0.001
**SBP (mmHg)**	101.43 ± 11.33	103.16 ± 11.98	99.96 ± 10.55	< 0.001
**DBP (mmHg)**	60.06 ± 7.41	60.51 ± 7.65	59.69 ± 7.20	0.149
**FPG (mg/dl)**	88.15 ± 5.67	88.87 ± 5.55	87.55 ± 5.71	0.002
**TC (mg/dl)**	182.18 ± 30.58	179.00 ± 30.27	184.85 ± 30.62	0.012
**TG (mg/dl)**	84.28 ± 42.46	78.38 ± 40.56	89.26 ± 43.43	0.001
**LDL (mg/dl)**	105.24 ± 27.03	102.79 ± 25.66	107.30 ± 28.00	0.028
**HDL (mg/dl)**	60.08 ± 11.95	60.53 ± 11.91	59.69 ± (11.99	0.358
**TC/HDL-C**	3.12 ± 0.69	3.03 ± 0.60	3.20 ± 0.75	0.002

Data are presented as means ± SD. Univariate analyses were calculated by t-test. BMI, body mass index; WC, waist circumference; SBP, systolic blood pressure; DBP, diastolic blood pressure; FPG, Fasting plasma glucose level; TC, total cholesterol level; TG, triglyceride level; LDL-C, low-density lipoprotein cholesterol level; HDL-C, high-density lipoprotein cholesterol level.

**Table 2:** Prevalence of pre-hypertension and hypertension by BMI for age and WC among school children

**Criteria**	**BMI for age and gender**				**p-value**	**WC**		**p-value**
	**Under weight**	**Normal**	**Over weight**	**Obese**		**< P**_**75**_	**≥ P**_**75**_	
Normal	62 (100.0)	375 (96.9)	102 (97.1)	106 (73.1)	< 0.001	508 (97.3)	131 (76.6)	< 0.001
Pre-hypertension	0 (0.0)	6 (1.6)	2 (1.9)	19 (13.1)		9 (1.7)	18 (10.5)	
Hypertension	0 (0.0)	6 (1.6)	1 (1.0)	20 (13.8)		5 (1.0)	22 (12.9)	

Data are presented as n (%) and were calculated by χ^2^ test. SBP, systolic blood pressure and DBP, diastolic blood pressure ≥ P_90_ but less than P_95_ was defined as pre-hypertension; ≥ P_95_ was defined as hypertension with boys and girls.

Table 3 shows that children with pre-HT and HT had significantly higher body weight, height, WC, BMI, SBP, DBP, TG, and TC/HDL-C levels but lower HDL-C levels than children with normotension. There was no significant difference in the mean of FPG, TC and LDL-C levels after controlling for gender and age.

**Table 3:** Age and sex-adjusted means of anthropometric and lipid measures by blood pressure among school children

**Variables**		**Blood pressure criteria**		**p-value**
	**Normotension (n = 639)**	**Pre-hypertension (n = 27)**	**Hypertension (n = 27)**	
	$\bar{X}$ **(SE)**	$\bar{X}$ **(SE)**	$\bar{X}$ **(SE)**	
**Weight (kg)**	37.76^a,b^ (0.42)	49.23^a^ (2.05)	53.47^b^ (2.05)	< 0.001
**Height (cm)**	140.61^b^ (0.28)	143.95 (1.35)	144.38^b^ (1.35)	0.002
**BMI (kg/m**^**2**^**)**	18.76^a,b^ (0.16)	23.43^a^ (0.79)	25.21^b^ (0.79)	< 0.001
**WC (cm)**	69.17^a,b^ (0.43)	82.36^a^ (2.11)	86.42^b^ (2.11)	< 0.001
**SBP (mmHg)**	99.64^a,b^ (0.36)	118.91^a,c^ (1.75)	126.20^b,c^ (1.75)	< 0.001
**DBP (mmHg)**	59.15^a,b^ (0.26)	70.00^a^ (1.28)	71.71^b^ (1.28)	< 0.001
**FPG (mg/dl)**	88.02 (0.22)	90.46 (1.07)	89.00 (1.07)	0.06
**TC (mg/dl)**	182.29 (1.21)	180.71 (5.88)	180.96 (5.87)	0.95
**TG (mg/dl)**	82.29^a,b^ (1.64)	105.46^a^ (8.02)	110.40^b^ (8.00)	< 0.001
**LDL-C (mg/dl)**	105.14 (1.07)	107.21 (5.20)	105.56 (5.19)	0.93
**HDL-C (mg/dl)**	60.69^a,b^ (0.47)	52.36^a^ (2.27)	53.23^b^ (2.27)	< 0.001
**TC/HDL-C**	3.09^a,b^ (0.03)	3.50^a^ (0.13)	3.50^b^ (0.13)	< 0.001

Values are presented as mean(SE). Mean difference was analyzed by ANCOVA adjusted age and gender.

Means in row with superscript letter differ as defined pos hoc analysis.

^a^The significant difference between normal (normotension) and pre-hypertension (p-value < 0.05).

^b^The significant difference between normal and hypertension (p-value < 0.05).

^c^The significant difference between pre-hypertension and hypertension (p-value < 0.05).

Table 4 presents the results from multiple regression analysis for association between BP and BMI/WC controlling for all covariates. FPG, BMI and WC were significantly associated with SBP and DBP. The association of high blood pressure and obesity status (BMI and WC) after controlling for sex, age, plasma glucose and lipid levels is shown in Table 5. Using the normal BMI for age as reference, the adjusted odds ratios for pre-HT and HT risk from obesity were 9.00 (95% CI, 3.20-25.31) and 10.60 (95% CI, 3.75-30.00) after adjusting for gender, age, plasma glucose and lipid values. Likewise, the adjusted odds ratios for pre-HT and HT for WC (abdominal obesity) alone were 6.20 (95% CI: 2.60-14.81) and 13.73 (95% CI: 4.85-38.83).

**Table 4:** Multiple regression for association between obesity/waist circumference and blood pressure including all covariates

**Variables**		**SBP**			**DBP**
	**Model 1 β (SE)**	**Model 2 β (SE)**	**Model 3 β (SE)**	**Model 4 β (SE)**	
Constant	50.98** (5.80)	44.00** (5.70)	34.05** (4.37)	30.74** (4.37)
Age	0.94** (0.30)	0.66* (0.29)	0.36 (0.23)	0.23 (0.23)
Sex	-1.37 (0.70)	-1.02 (0.68)	1.44 (0.52)	0.30 (0.52)
FPG	0.15* (0.06)	0.13* (0.06)	0.11* (0.05)	0.10* (0.05)
TG	0.01 (0.01)	0.01 (0.01)	0.01 (0.01)	0.01 (0.01)
LDL-C	-0.01 (0.02)	-0.01 (0.02)	0.04 (0.01)	0.003 (0.01)
TC/HDL-C	-0.21 (0.88)	-0.35 (0.85)	-0.35 (0.85)	-0.91 (0.65)
BMI	1.47** (0.09)	-	0.71** (0.06)	-
WC	-	0.58** (0.03)	-	0.28** (0.02)
R^2^	0.41	0.44	0.22	0.23

Model 1; age, sex, FPG, TG, LDL-C, TC/HDL-C and BMI.

Model 2; age, sex, FPG, TG, LDL-C, TC/HDL-C and WC.

Model 3; age, sex, FPG, TG, LDL-C, TC/HDL-C and BMI.

Model 4; age, sex, FPG, TG, LDL-C, TC/HDL-C and WC.

*p-value < 0.05; **p-value < 0.01.

**Table 5:** Multinomial logistic regression adjusted odds ratios for association between BMI/ WC and blood pressure

**Variables**	**Pre-hypertension**	**Hypertension**
**BMI for age and gender**		
- Normal	Reference group	Reference group
- Overweight	1.05 (0.20-5.50)	0.50 (0.06-4.42)
- Obesity	9.00** (3.20-25.31)	10.60** (3.75-30.00)
**WC**		
- < P75	Reference group	Reference group
- ≥ P75	6.20** (2.60-14.81)	13.73** (4.85-38.83)

Multinomial logistic regression was computed for high blood pressure risk factors controlling age, sex, plasma lipid (TG and TC/HDL-C) and glucose (FPG) levels. Values were reported as odd ratio (OR) and 95% confidence intervals (95% CI). **p-value < 0.01.

## Discussion

Our findings revealed that anthropometric measures (weight, height, BMI and WC) and biochemical measurements of TG and TC/HDL levels among children with pre-HT and HT were significantly higher than those among children with normotension. This indicated that obese children showed a significantly higher prevalence of pre-HT and HT than non-obese children.

Our mean prevalence for obesity at 20.9% in two schools was higher than the national prevalence reported by the Thailand National Health examination survey as 6.7% in 2001 and as 8.7% in 2010 [7], with the highest regional prevalence of 14.8% in Bangkok [8]. The latter was similar to the finding of the Shanghai CDC that reported the prevalence of child obesity as 13.5% in 2009 [19]. Our higher prevalence of obesity than the Thai national prevalence may have been the result of using the CDC growth chart, since Thai children categorized as obese had greater weight but relatively shorter stature when compared to American children of the same age and sex. This would result in relatively high BMI or obesity rates. On the other hand, the mean prevalence of obesity (20.9%) in our study group was lower than that reported for children from the US at 31.0% [20] but the same as that reported by the South Carolina Pediatric Practice Research Network at 21.7% [21].

Our study revealed that obesity prevalence for boys was over 30% while that for girls was almost three times less at 12%. Similar results have been reported from China [22] and Taiwan [23]. We did not collect data amenable to analysis for a possible explanation regarding this difference. Neither was an explanation given in the cited studies from China and Taiwan. Therefore, we must resort solely to speculation regarding possibilities. In a previous report [2], it was explained that the overall increase in obesity in Thai children may have been encouraged by a traditional positive attitude that overweight children are healthy and cute and by subsequent overindulgence, especially in small families. However, these are overall factors that may not apply evenly to boys and girls. For example, it is possible that girls are subjected to social and media pressure to be slim and shapely while boys are not, and this may result in some measure of weight control motivation that is present in girls but not in boys. To obtain an answer to such speculation, a more sophisticated motivational study than ours would be required.

WC is an indicator of distribution of abdominal or visceral fat, and also is an indicator for insulin resistance, type 2 diabetes, dyslipidemia and cardiovascular disease in the form of high blood pressure in childhood and adulthood [24]. Because the WC cutoff for abdominal obesity varies among different populations [25] and because a Thai reference standard is still lacking, we used a cutoff at P_75_ for screening cardiovascular risks in our children. This cutoff at P_75_ is similar to one of the cutoffs used for abdominal obesity screening for cardiovascular risks in Chinese children [25].

The high prevalence of overweight/obesity among school-aged children in this study might be due to less physical activity and higher consumption of more unhealthy, high-fat foods, sweetened beverages and salty snacks and less milk, fruits and vegetables than recommended [2,3,26].Various studies have shown that dietary intake, physical activity and self-discipline are major factors influencing obesity and high blood pressure [1-3,27].

Lipid profiles of girls were significantly higher than those of boys, as reported in other studies [28,29] where this was suggested to result primarily from poor eating habits and low physical activity and to be independent of nutritional status. In a previous study [3] it was reported that a high intake of cholesterol and saturated fatty acids originated from popular diets such as fried chicken, sausages and cakes that are high in saturated fatty acids and cholesterol that influence TC and LDL-C levels. In addition to fats, favorite snacks tend to be high also in carbohydrates and salt. Excess carbohydrates can be changed to TG [30] and this may be reflected in the higher TG levels.

Obese children showed a statistically significant higher prevalence of pre-hypertension and hypertension than non-obese children, and this was similar to findings from other studies [9,19]. The finding of HT prevalence at 13.8% in the present study was lower than that found in Canadian and Chinese children (19.5% and 25.6%, respectively). The mean levels of FPG, TC and LDL-C were not significantly different among the three BP groups, a result similar to that reported in a study by Boyd *et al*. [10]. Plasma glucose, obesity and WC (abdominal obesity) alone were associated with SBP and DBP by multiple regression analysis, but there was no association of lipid profiles with SBP and DBP. However, previous studies have reported that plasma glucose and lipids are correlated with blood pressure linked to metabolic syndromes [10,31].

Overall, our study revealed that there were positive associations between high SBP/DBP and BMI or WC alone after adjusting for age, gender, FPG and lipids levels. These results were similar to those of previous studies indicating that high blood pressure increased with increasing BMI and WC after adjusting for age and sex [19,32]. These findings suggest that obese children are at higher risk of having high blood pressure than normal children. Therefore, obese children should be routinely screened for blood pressure and other coexisting cardiovascular risk factors including lipid profile [3,33]. In addition, healthy lifestyles should be encouraged by schools and at home since prevention in childhood can help to avoid undesirable health consequences in the future [2,4].

Limitations of our study include of the following. First, our research was a cross-sectional study that cannot explain the causal relationships between high blood pressure and obesity. Second, blood pressure was measured on a single occasion and would be more accurate if confirmed on several occasions. Finally, the sample size for the obese group was quite small when compared with the reference group, leading to the wide range of 95% CI. Hence, a larger study to confirm the precision of the association might be needed.

## Conclusions

This study revealed that obesity was positively and significantly associated with higher blood pressure. Therefore, development of healthy lifestyle practices should be encouraged from childhood in schools and at home to prevent and control future undesirable cardiovascular consequences.

## Abbreviations

BMI: Body mass index; CI: Confidence Interval; DBP: Diastolic blood pressure; FPG: Fasting plasma glucose; HDL-C: High density lipoprotein-cholesterol; HT: Hypertension; LDL-C: Low density lipoprotein-cholesterol; OBEC: Office of the Basic Education Commission; OR: Odds ratios; P: Percentile; pre-HT: Pre-hypertension; SBP: Systolic blood pressure; SD: Standard deviation; TC: Total cholesterol; TG: Triglyceride; WC: Waist circumference.

## Competing interests

The authors declare that they have no competing interests.

## Authors’ contributions

PS was the principal investigator. CS was the project manager, initiated the concept of the study, obtained research grant support, coordinated the research, assigned individual roles of participants and was responsible for overall supervision. PS, WA, CH and CS made contributions to the design of the study. PS was involved in the literature review and data collection. PS and WA made contributions in analysis and interpretation of the data, and drafting of the manuscript. CS revised the content and approved the manuscript. All authors read and approved the final manuscript.

## Pre-publication history

The pre-publication history for this paper can be accessed here:

http://www.biomedcentral.com/1471-2458/14/729/prepub

## Supplementary Material

Additional file 1 **1.1 Child nutritional status.** 1.2 Child blood pressure classification. Click here for file

Additional file 2 **The 75**^**th **^**percentile for age-and sex- specific waist circumference (WC) cut-off points.** Click here for file
