# Supplementary material for: The association between obesity and blood pressure in Thai public school children
Source: BMC Public Health. 2014 Jul 18;14:729. doi: 10.1186/1471-2458-14-729 (PMC4223408; doi:10.1186/1471-2458-14-729)
Supplement: Additional file 1 — 1.1 Child nutritional status. 1.2 Child blood pressure classification. [file 1471-2458-14-729-S1.docx]

**Additional file 1**

**1.1 Child nutritional status**

The data of nutritional status (age and sex specific BMI percentiles) for each child were calculated based on the 2000 Centers for Disease Control and Prevention growth charts [13].

BMI was estimated according to the known formula (kg/m^2^) and the study population was classified into three categories as 1) normal, 2) risk for overweight and 3) obese using the 2000 CDC growth charts according to age and gender.

Computation of BMI percentile for gender and age was calculated from the following equation:

X = M (1 + LSZ) ^(1/L)^, L ≠ 0

L, M and S are the values from the appropriate table corresponding to the age in months of the child. The LMS parameters are the power in the Box-Cox transformation (L), the median (M) and the generalized coefficient of variation (S) used to obtain the z-score (Z) and corresponding percentile for a given measurement (X; X=BMI value by kg/m^2^ unit) [14]. BMI was categorized by using the 2000 Centers for Disease Control and Prevention growth charts for boys and girls aged 2 to 20 (underweight: <5^th^ percentile; normal weight: 5^th^-85^th^ percentiles; overweight: 85^th^-95^th^ percentiles; obese: ≥95^th^ percentile) [14].

**1.2 Child blood pressure classification**

We used the charts provided by the fourth report on diagnosis, evaluation and treatment of high blood pressure in children and adolescents (**Pediatrics 2004; 114: 555-576**) and classified the study population into three categories as 1) Normal, 2) High-normal and 3) High blood pressure, as recommended.

Computation of BP percentiles according to gender, age and height was based on National High Blood Pressure Education Program Working Group on high BP in children and adolescents [19]. Refer to the LMS transformation equation above. We converted the height to a height Z score relative to boys and girls of the same age, and then computed the expected systolic blood pressure (SBP) and diastolic blood pressure (DBP) denoted by µ for boys and girls of age and height according to the following equation:


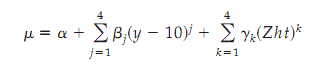


The computation of BP percentiles in this study was documented in BP percentile for arbitrary gender, age and height [19]. Blood pressure was classified for SBP and DBP as 1) normal <90^th^ percentile, 2) pre-hypertension (pre-HT) as >90^th^ percentile but <95^th^ percentile and 3) hypertension (HT) as ≥95^th^ percentile [19].
