# Supplementary material for: The association between obesity and blood pressure in Thai public school children
Source: BMC Public Health. 2014 Jul 18;14:729. doi: 10.1186/1471-2458-14-729 (PMC4223408; doi:10.1186/1471-2458-14-729)
Supplement: Additional file 2 — The 75 th percentile for age-and sex- specific waist circumference (WC) cut-off points. [file 1471-2458-14-729-S2.docx]

**Additional file 2: The 75^th^ percentile for age-and sex- specific waist circumference (WC) cut-off points.**

| **Age group (years)** | **WC for boys (cm)** | **WC for girls (cm)** |
| --- | --- | --- |
| 8  (58 boys, and 51 girls) | 76.6 | 68.2 |
| 9  (72 boys, and 92 girls) | 79.5 | 73.7 |
| 10  (75 boys, and 80 girls) | 87.4 | 76.1 |
| 11  (102 boys, and 142 girls) | 85.3 | 76.1 |
| 12  (10 boys, and 11 girls) | 84.1 | 74.6 |
